# Camptothecin enhances the anti-tumor effect of low-dose apatinib combined with PD-1 inhibitor on hepatocellular carcinoma

**DOI:** 10.1038/s41598-024-57874-6

**Published:** 2024-03-26

**Authors:** Hankang Wang, Congcong Gao, Xiaodong Li, Feng Chen, Guijie Li

**Affiliations:** 1 Department of Radiology, The First Affiliated Hospital of Shandong First Medical University, Jinan, Shandong 250000 People’s Republic of China; 2 Jinan Center for Disease Control and Prevention, Jinan, Shandong 250000 People’s Republic of China; 3 Department of Radiology, The First Affiliated Hospital of Shandong First Medical University, Jinan, Shandong 250000 People’s Republic of China; 4 Department of Radiology, The First Affiliated Hospital of Shandong First Medical University and Shandong Provincial Qianfoshan Hospital, 16766 Jingshi Road, Lixia, Jinan, Shandong 250014 People’s Republic of China

**Keywords:** Hepatocellular carcinoma, Camptothecin, Apatinib, PD-1 inhibitor, Nrf2, Cancer, Immunology, Diseases, Oncology, Pathogenesis

## Abstract

Apatinib has been shown to apply to a variety of solid tumors, including advanced hepatocellular carcinoma. Preclinical and preliminary clinical results confirmed the synergistic antitumor effects of apatinib in combination with anti-programmed death-1 (PD-1) inhibitors. In this study, we investigated camptothecin (CPT) enhances the anti-tumor effect of low-dose apatinib combined with PD-1 inhibitor on hepatocellular carcinoma. CPT combined with a PD-1 inhibitor enhances the anti-tumor effects of low-dose apatinib in hepatocellular carcinoma which was evaluated in making use of the H22 mouse model (n = 32), which was divided into four groups. Immunohistochemical staining and western blotting were used to detect nuclear factor erythroid 2-related factor 2 (Nrf2) as well as sequestosome 1 (p62), vascular endothelial growth factor A (VEGFA), vascular endothelial growth factor receptor 2 (VEGFR2), PD-1, and programmed cell death ligand 1 (PD-L1). The results showed that the average size of the tumor of the combination group (Group D) was significantly less than that of the apatinib + PD-1 inhibitor group (Group C). The expression levels of Nrf2, p62, VEGFA, VEGFR2, PD-1, and PD-L1 in the apatinib + PD-1 inhibitor group(Group C) were lower than those in the control group (Group A) (*P* < 0.05). The expression levels of these genes in the apatinib + PD-1 inhibitor group (Group C) were significantly lower in the combination group (Group D) (*P* < 0.05). There was no obvious difference in body weight and liver and kidney functions between the four groups of mice. In conclusion, CPT improves the anti-tumor effect of low-dose apatinib combined with PD-1 inhibitor on hepatocellular carcinoma

## Introduction

Liver cancer is the eighth most common and third leading cause of cancer death, HCC accounts for 80% of all liver cancers^1^. HCC patients often experience recurrence of tumors, which account for approximately 70–80% of HCC-related deaths^2,3^. Apatinib is an orally administered small-molecule tyrosine kinase inhibitor (TKI) that very selectively blocks VEGFR2 and inhibits VEGF-mediated endothelial cell proliferation and migration^4^. VEGFR2 is thought to be the primary receptor for pro-angiogenic signaling downstream of VEGFA. Apatinib also promotes reactive oxygen species (ROS) production and inhibits the expression of Nrf2 and p62, leading to autophagy and apoptosis. Apatinib induces autophagy and apoptosis in tumor cells by regulating the VEGFR2/PD-L1 and ROS/Nrf2/p62 signaling pathways^5^. Apatinib inhibits ovarian cancer cell growth by promoting apoptosis and autophagy in Nrf2 and p62^6^. Other studies have also reported that apatinib inhibits cell growth and metastasis and promotes apoptosis by regulating autophagy in a variety of human cancers^7,8^. Currently, a large number of studies have shown favorable therapeutic effects of apatinib in a variety of solid tumors^9–13^. Compared with other VEGFR TKIs, apatinib is easy to administer and less economically burdensome, making it an interesting emerging antiangiogenic therapy.

Recently, immunotherapy has brought revolutionary changes to cancer treatment, effectively controlling previously incurable highly invasive cancers. Antibodies against PD-1 and its ligand, PD-L1, are widely used to treat malignant tumors, including HCC^14,15^. Immune checkpoint inhibitor therapies represent a great method in the treatment of a variety of solid tumors by inhibiting the interaction between PD1, mainly expressed on activated CD8 + T cells, and PD-L1^16,17^. PD-1 inhibitor reactivates damaged T cells and restores its ability to kill tumor cells^18,19^. However, the result of single-agent PD-1 inhibitor is 20–40% in a variety of solid tumors^19,20^. Roger et al.^21^ found that the vasoproliferative tumor microenvironment (TME) is strongly associated with resistance to PD-1 inhibitor. Hypoxia, pro-angiogenesis and epithelial-mesenchymal transition are correlates of PD-1 resistance, the most important of which is the high expression of VEGFA^21^.

Nrf2 is encoded by the nuclear factor erythroid-derived 2-like 2 (NFE2L2) gene^22–26^. In normal conditions, Kelch-like ECH-associated protein (Keap1) binds to Nrf2, causing it to be degraded by proteasomes in the cytoplasm^22–28^. The antioxidant response element-mediated cytoprotective proteins include antioxidant enzymes, stress-responsive proteins, metal-binding proteins, drug-metabolizing enzymes, and drug-transport proteins^26^. Carcinogenesis is also a novel function of Nrf2^22,23,26^. Activation of Nrf2 protects cells from oncogenic chemicals for a short period of time^29–32^.

CPT is a natural alkaloid that is a potent antitumor agent. It binds to DNA topoisomerase I to inhibit DNA replication^30,33,34^. Previous studies by the authors confirmed that CPT is an Nrf2 inhibitor that is effective at lower concentrations, thereby reducing drug toxicity^30,34^. CPT can be used in combination to treat HCC by modulating Nrf2 levels^30^. Based on the above studies, we hypothesized that CPT could act by inhibiting the NRF2 pathway, synergizing with apatinib’s inhibition of the NRF2 pathway, making it useful at a lower dose in combination with PD-1 inhibitors. In this study, we established a mouse liver tumor H22 model and compared the effects of the low-dose apatinib + PD-1 inhibitor group with the combination group on TME to test this hypothesis.

## Materials and methods

### H22 HCC model

Animal experiments were conducted in accordance with the ARRIVE guidelines (Approval No. S0007) approved by the Animal Protection and Utilization Committee of the First Hospital of Shandong First Medical University (Jinan). Confirm that all experiments are conducted in accordance with relevant guidelines and regulations. The mice were purchased by Beijing Viton Lihua Company. H22 tumors were provided by the laboratory of Qianfoshan Hospital. CPT, Aptinib and PD-1 inhibitor were purchased from Jinan Shengshi Biotechnology Co. H22 tumors were provided by the laboratory of Qianfoshan Hospital.

Male BALB/c mice (3–5 weeks old, 18–22 g) were divided into two batches (n = 20/batch), housed under standard animal husbandry conditions with room temperature, sufficient air, 12/12 h light/dark cycles, and permitted to use sterilized water and feed ad libitum. Mice were subcutaneously inoculated with H22 cells (1 × 10^6^/200 µl saline).

When the tumors reached the size of soybean grains, a caliper was used to measure the volume of the tumors, with tumor volume (mm^3^) = π/6 × length × width^35^. Thirty-two out of 40 mice with similar tumor sizes were selected for enrollment, while the remaining 8 mice did not meet the criteria for tumor size. The criteria for tumor size around 7–8 mm. A total of 32 mice survived in good condition until the end of the experiment and were euthanized by sodium pentobarbital injection. Mice were euthanized by slow intraperitoneal injection of 2% sodium pentobarbital (150–200 mg/kg) until death. Finally, tumors were dissected after euthanasia and weighed after tumor removal to analyze tumor volume and body weight. Analyses were performed using GraphPad Prism software (version 8; GraphPad Software, Inc.). 32 mice with similar tumor size were randomly divided into 4 groups (group A) (saline), CPT group (group B) (3 mg/kg CPT), apatinib + PD-1 inhibitor group (group C) (60 mg/kg apatinib + 10 mg/kg PD-1 inhibitor), and apatinib + PD-1 inhibitor combined with CPT group (group D) (60 mg/kg apatinib + 10 mg/kg PD-1 inhibitor + 3 mg/kg CPT)^36–38^. CPT was injected intraperitoneally every 3 days, and apatinib was administered daily by gavage, while PD-1 inhibitor was injected intraperitoneally every 3 days. A total of 40 mouse models were established, of which 32 were eligible for enrollment; the 8 animals not enrolled were euthanized by sodium pentobarbital injection.

All animal welfare was taken into account, including minimizing pain and suffering, the use of painkillers or anesthetics, or special housing conditions. The experimental duration of the mouse model was 30 d. After the completion of the experimental objectives, the animals were treated in a scientific and humane manner to minimize their panic and suffering, and euthanasia was performed gently and quickly. By observing respiration, cardiac arrest, pupil, nerve reflexes and other indicators, the death was comprehensively judged, and it was confirmed that the experimental animals had died.

### Immunohistochemical staining

The H22 tumor were fixed in polyformaldehyde (4%) and embedded in paraffin wax at room temperature. Sections of 4‑µm thickness were cut, mounted on charged glass slides and then stained with haematoxylin and eosin at room temperature. Briefly, hema‑toxylin was added to the sections for 10 min. Then, 1% acid ethanol reagent was used to differentiate for 1 min. Then, the blue returning liquid promoted the nucleus to return blue, and then the eosin solution was incubated with sections for 3 min. Finally, the sections were dehydrated and fixed with neutral balsam. Paraffin sections (4 µm) of H22 tumors were deparaffinized with xylene and rehydrated with a descending ethanol series. The sections were blocked with bovine serum albumin for 30 min at 37 °C and covered with anti-Nrf2 antibody (1:1000; cat. no. GB113808), VEGFA (1:200; cat. no. 19003-1-AP), p62 (1:1000; cat. no. GB11239-1), PD-1 (1:1000; cat. no. GB12338), cMyc (1:200; cat. no. GB13076), TGF-β (1:500; cat. no. GB11179) CD4 (1:500; cat. no. GB15064), and CD8 (1:500; cat. no. GB15068), overnight at 4 °C. Antibodies against Nrf2, VEGFA, p62, PD-1, cMyc, TGF-β, CD4, and CD8 were obtained from Wuhan Servicebio Technology Co. Sections were then incubated with HRP-labeled goat anti-mouse IgG solution (cat. no. G1214-100UL; from Wuhan Servicebio Technology Co., Ltd.) diluted at a 1:200 dilution for 30 min at 37 °C, and next, DAB substrate was added. Cell nuclei were counterstained with hematoxylin. Images were captured under a light microscope. Staining was visualized using Image-ProPlus 6 software (Media Cybernetics, Inc.) and integrated optical density/area values were used to determine protein expression levels in the tumors.

### Western blotting

H22 tumors were pulverized in RIPA (Wuhan Servicebio Technology Co., Ltd.) buffer with 1 mM PMSF on ice and then centrifuged as previously described^26^. Protein concentration was determined using Bicinchoninic acid (BCA). Protein samples (15 µg samples per lane) were loaded into 30% SDS—PAGE gels and transferred to PVDF membranes. The membranes were blocked with 3% bovine serum albumin for 1 h at room temperature and then incubated at 4 °C with the following primary antibodies (obtained from Wuhan Servicebio Technology Co., Ltd.) incubated overnight at 4 °C with the following primary antibody: Nrf2 (1:1000; cat. no. GB113808), P62 (1:1000; cat. no. GB11531), VEGFA (1:1000; cat. no. GB11034B), VEGFR2 (1:1000; cat. no. GB11190; from Wuhan Servicebio Technology Co., Ltd.), p-VEGFR2 (1:1000; cat. No AF3279), PD-1 (1:1000; cat. no. GB11338), PD-L1 (1:1000; cat. no. GB11339A), CD69 (1:1000; cat. no. GB115670), IL-6 (1:1000; cat. no GB11117) and IFN-γ (1:1000; cat. no. MM700B). The membrane was then incubated with HRP-labeled goat anti-mouse IgG solution at a dilution of 1:5000 for 1 h at room temperature. Finally, the membranes were covered with enhanced chemiluminescence (ECL) substrate and scanned. ECL substrate was obtained from Merck Millipore. Quantification of the results normalized to β-actin was conducted using Image J software (version 1.8.0.345; National Institutes of Health).

### Serum biochemistry

The health and behavior of H22 mice model were monitored every day after H22 cells were inoculated subcutaneously into the mice. Blood was collected via the retro‑orbital sinus and was centrifuged at 4 °C, 1000×*g* for 5 min. Aspartate aminotransferase, alanine aminotransferase, blood urea nitrogen, creatinine and total bilirubin levels were measured using an automatic biochemical analyzer.

### Statistical analysis

Analyses were performed using GraphPad Prism software (version 8; GraphPad Software, Inc.). Data are presented as the mean ± SD. Comparisons between groups were performed using one‑way ANOVA with the post hoc test Tukey’s multiple comparison test. *P* < 0.05 was considered as indicating a statistically significant difference (Supplementary File S1).

### Ethics approval and consent to participate

All animal experiments were performed according to the ARRIVE guidelines approved (approval no. S0007) by the Animal Care and Use Committee of the First Hospital of Shandong First Medical University (Jinan, China).

## Results

### CPT enhances low-dose apatinib sensitivity in HCC by inhibiting the Nrf2/p62 pathway

**Figure 1:**
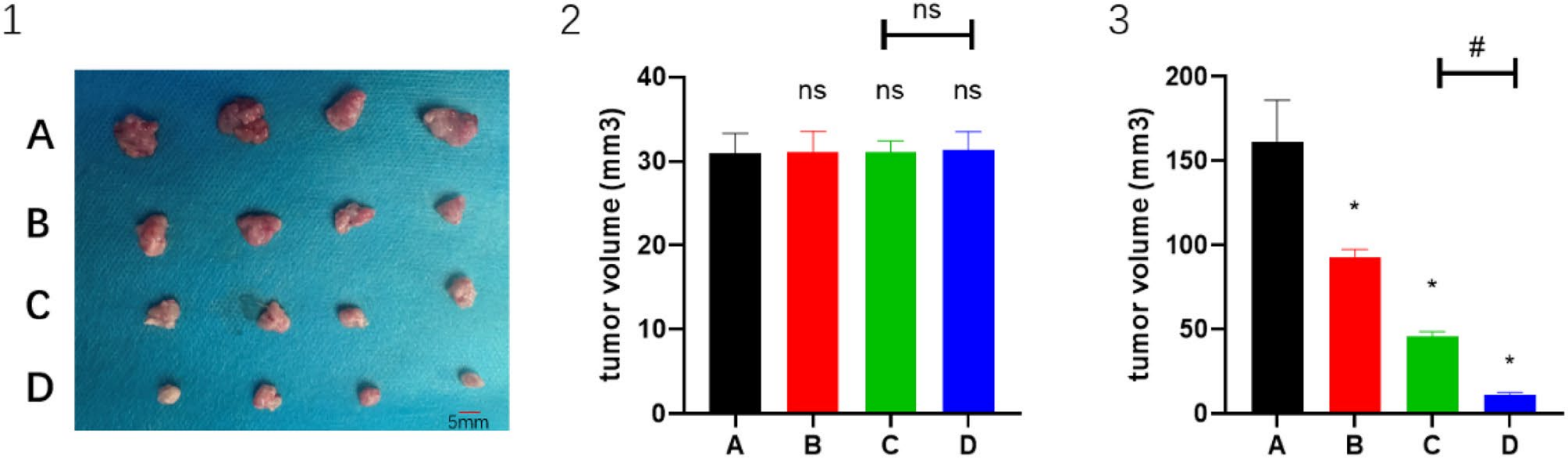
The effects of the combination of apatinib and PD-1 inhibitor and the combination of CPT in H22 models (1). The tumor volumes before (2) and after (3) treatment in A, B, C and D groups (n = 8). *P < 0.05 and ^#^*P* < 0.05.

When apatinib, PD-1 inhibitor, and CPT were administered, Nrf2 protein in mouse H22 tumor tissues was reduced and tumor growth was also inhibited after inhibiting Nrf2 protein expression. The effects of the combination of apatinib and PD-1 inhibitor and the combination of CPT are shown (Fig. 1(1)). The pre-treatment tumor volumes were 30.958 ± 2.315 mm^3^, 31.087 ± 2.470 mm^3^, 31.154 ± 1.251 mm^3^, 31.416 ± 2.113 mm^3^ (*P* ≥ 0.05) in groups A, B, C, and D, respectively (Fig. 1). Tumor volumes after treatment were 161.531 ± 24.616 mm^3^, 92.285 ± 5.353 mm^3^, 45.684 ± 2.959 mm^3^, 10.865 ± 1.552 mm^3^. The difference between groups A and C and groups C and D was statistically significant (*P* < 0.05) (Fig. 1(2,3)). This indicated that apatinib combined with PD-1 inhibitor inhibited tumor growth, and this inhibitory effect was even more pronounced after the combined administration of CPT.

**Figure 2:**
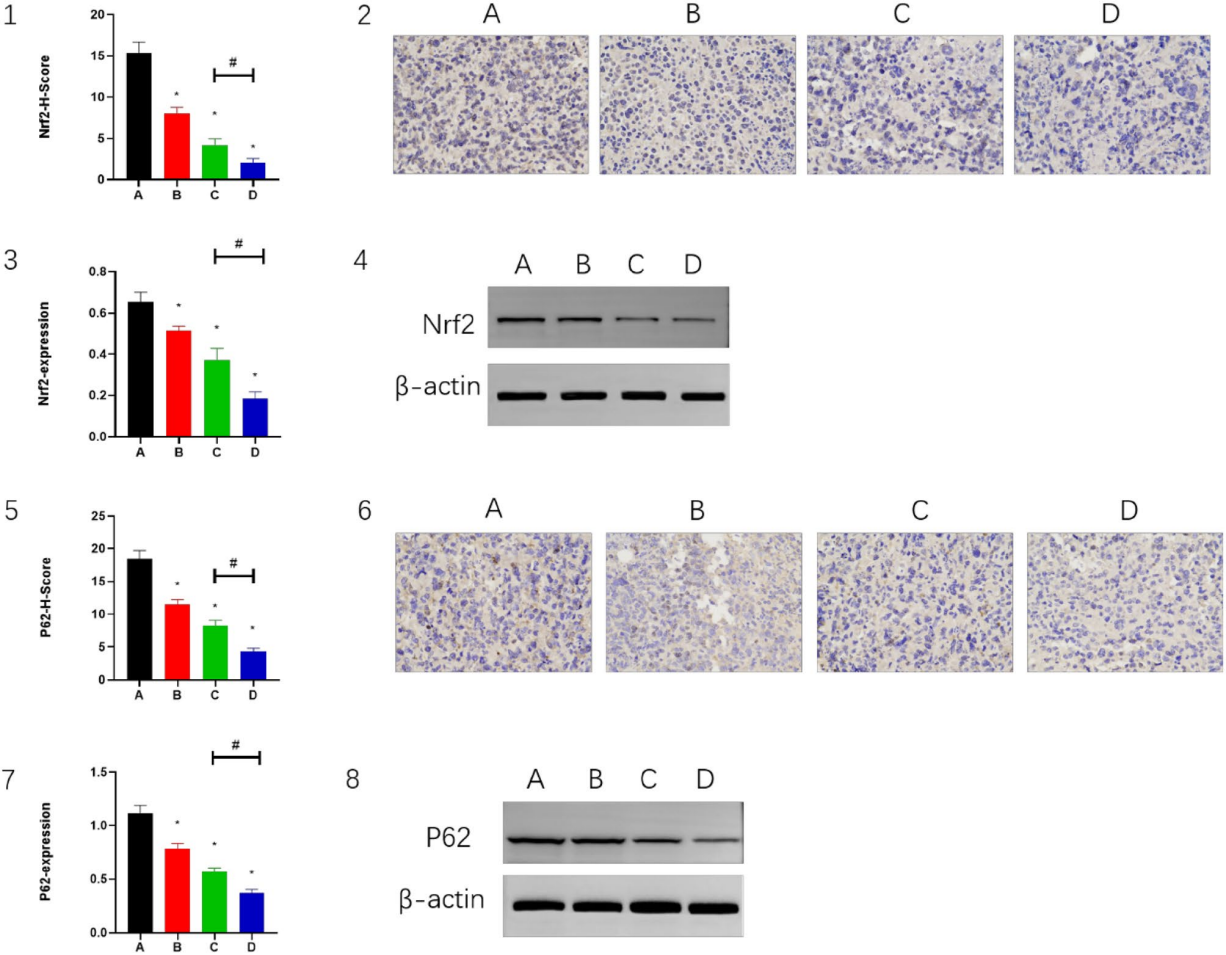
The Nrf2 expressed in (2) IHC staining and (4) WB. The P62 expressed in (6) IHC staining and (8) WB. Magnification, 400×. β-actin was the internal control of WB. Panels 1, 3, 5 and 7 demonstrate the statistical analysis for IHC staining and WB. *P < 0.05 and ^#^*P* < 0.05.

To detect the effects of apatinib, PD-1 inhibitor and CPT on the regulation of Nrf2 and p62 in vivo, western blotting and IHC staining were performed on mouse H22 tumor tissues. Treatment down-regulated Nrf2 expression in group B (*P* < 0.05) and decreased Nrf2 expression in group C (*P* < 0.05) compared with group A. However, Nrf2 expression was significantly decreased in group D (*P* < 0.05) compared with groups C. IHC and western blotting yielded comparable results (Fig. 2(1–4)). In addition, p62 was also affected in a similar manner (Fig. 2(5–8)). Apatinib combined with PD-1 inhibitor had an inhibitory effect on Nrf2 and p62, and the inhibition was more pronounced after the combined administration of CPT, suggesting that CPT could enhance HCC sensitivity to apatinib.

### Combination of CPT, apatinib, and PD-1 inhibitor inhibits angiogenesis

**Figure 3:**
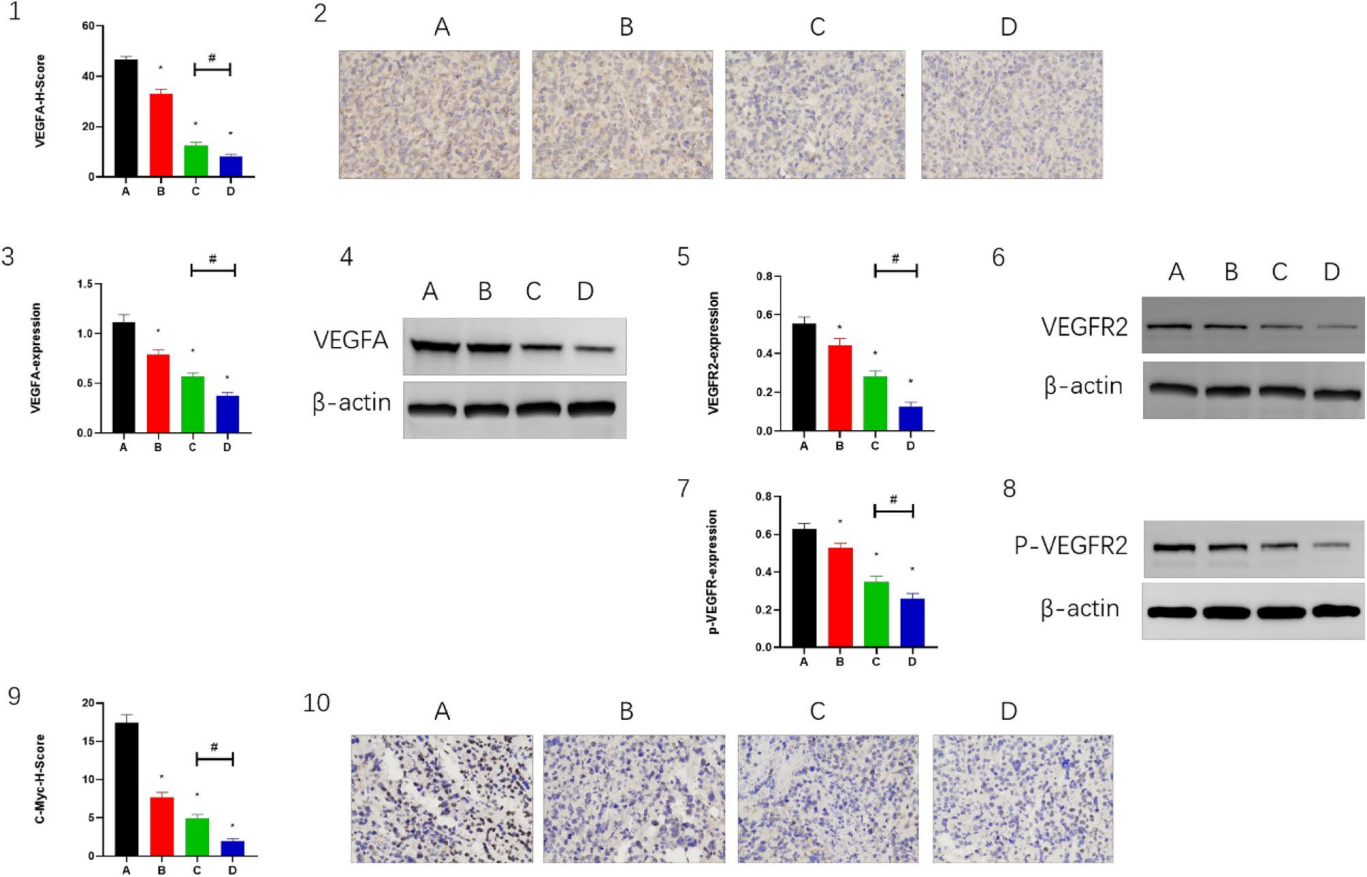
The VEGFA expressed in (2) IHC staining and (4) WB. The VEGFR2 expressed in (6) WB in H22 models. The p-VEGFR2 expressed in (8) WB in H22 models.The c-Myc expressed in (10) IHC staining. Magnification, 400×. β-actin was the internal control of WB. Panels 1, 3, 5, 7 and 9 demonstrate the statistical analysis for IHC staining and WB. *P < 0.05 and ^#^*P* < 0.05.

In the mouse H22 tumor model, the expression levels of VEGF A, VEGFR2 and p-VEGFR2 were lower in groups B and C than in group A (*P* < 0.05), whereas in group D, the expression levels of VEGFA and VEGFR2 were significantly lower than in groups A and C (*P* < 0.05) (Fig. 3(1–8)). Similarly, the downstream target of VEGFR2, c-Myc, showed similar changes (Fig. 3(9,10)). This indicated that apatinib combined with PD-1 inhibitor could inhibit tumor angiogenesis by inhibiting the expression of VEGFA and VEGFR2, and then c-Myc, and the inhibitory effect was even more evident with the combined administration of CPT.

### Combination of CPT, apatinib and PD-1 inhibitor improves tumor microenvironment

**Figure 4:**
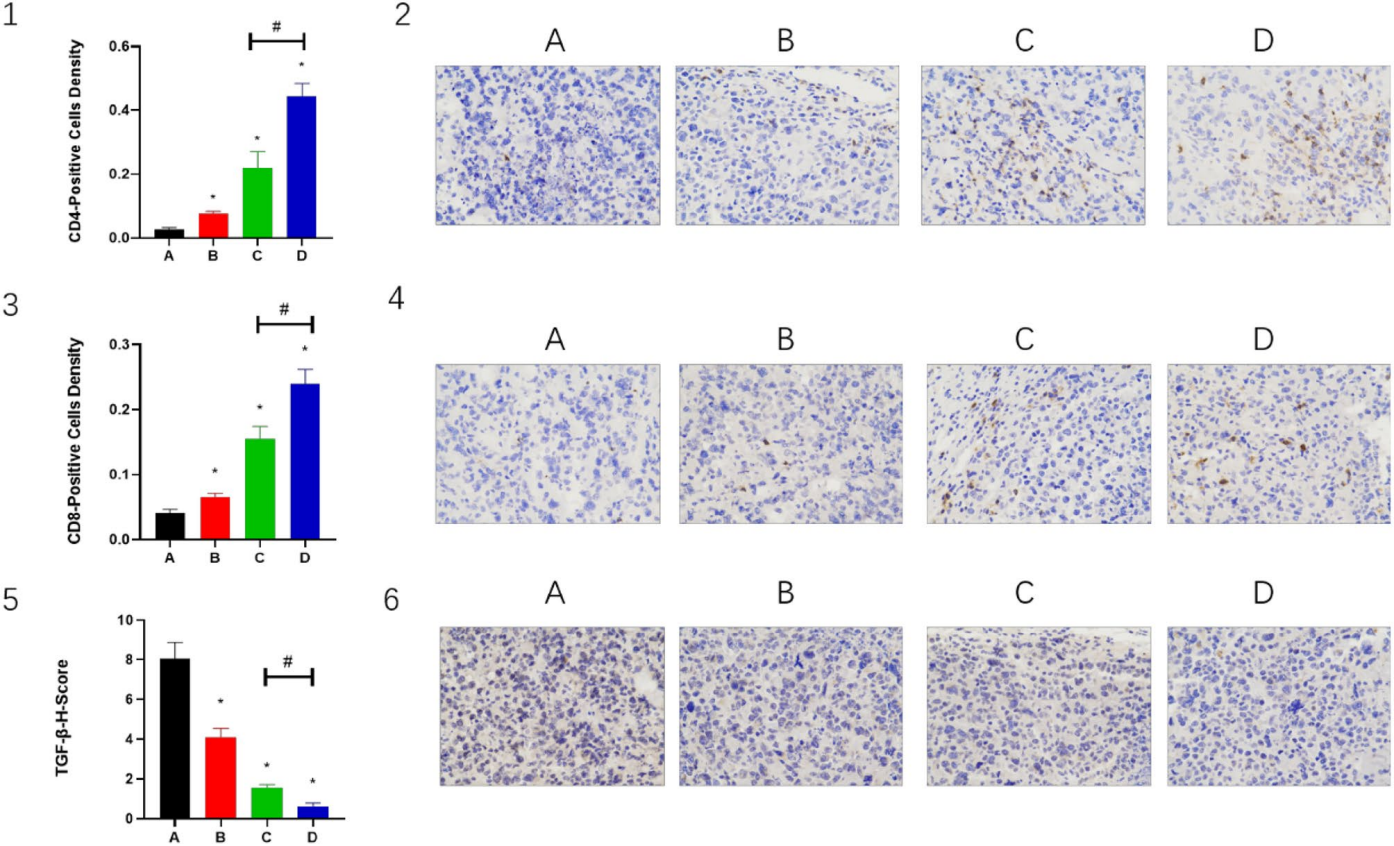
The CD4, CD8 and TGF-β expressed in (2, 4 and 6) IHC staining. Magnification, 400×. Panels 1, 3 and 5 demonstrate the statistical analysis for IHC staining. *P < 0.05 and ^#^*P* < 0.05.

**Figure 5:**
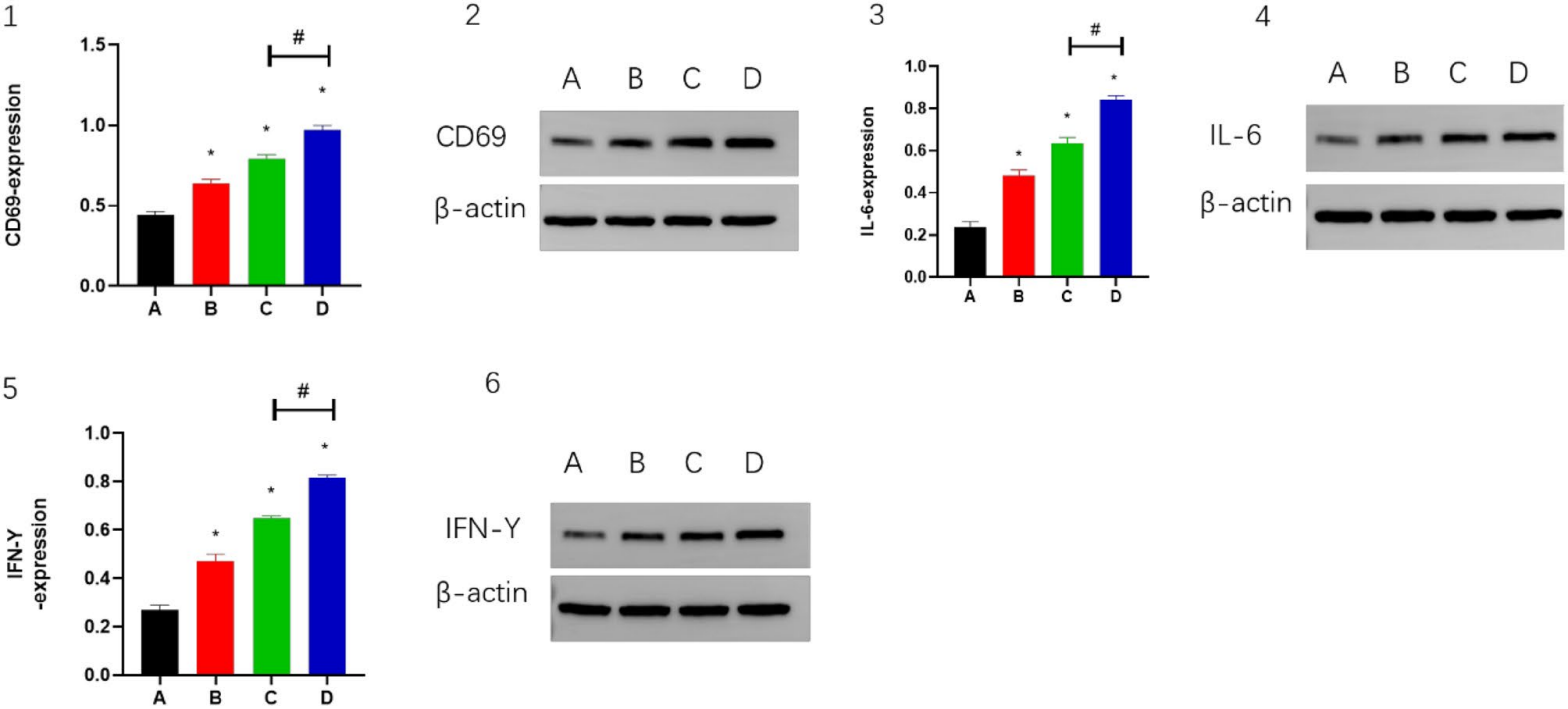
The CD69, IL-6 and IFN-γ expressed in (2, 4 and 6) WB. β-actin was the internal control of WB. Panels 1, 3 and 5 demonstrate the statistical analysis for WB. *P < 0.05 and ^#^*P* < 0.05.

**Figure 6:**
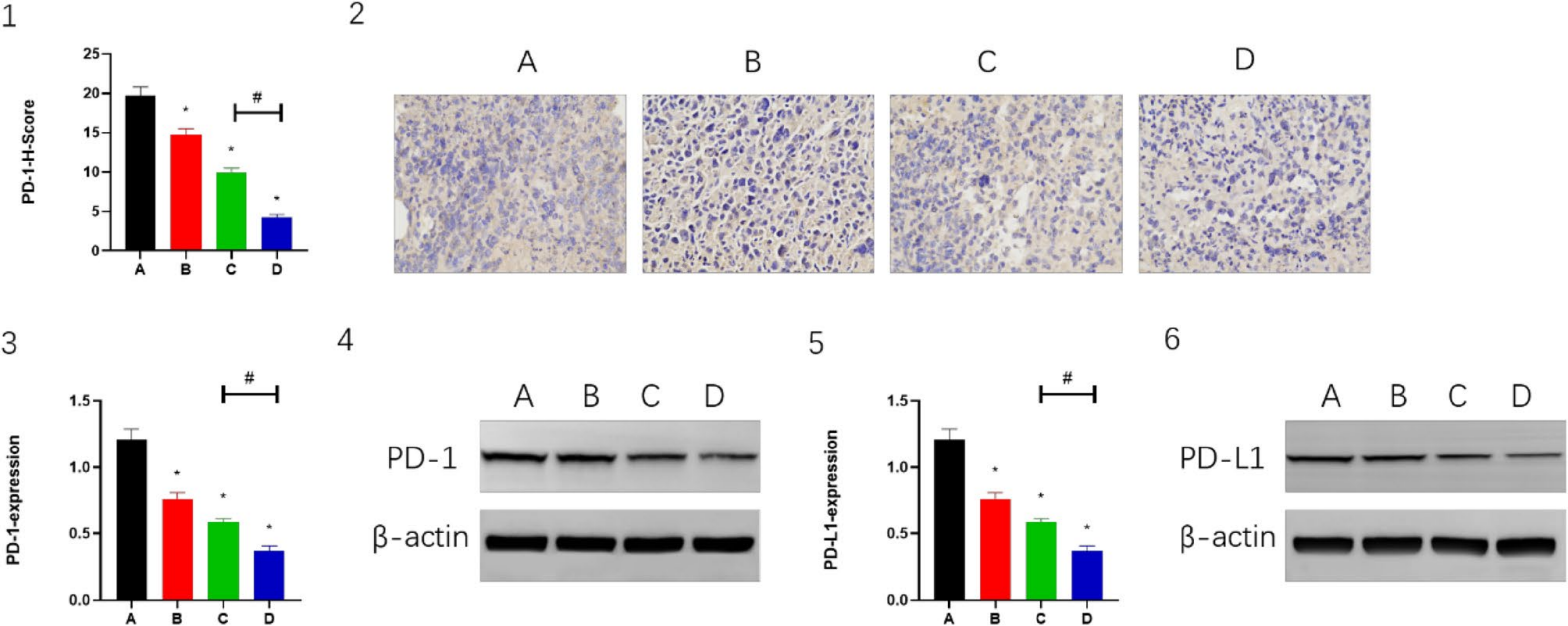
The PD-1 expressed in (2) IHC staining and (4) WB. The PD-L1 expressed in (6) WB. Magnification, 400×. β-actin was the internal control of WB. Panels 1, 3 and 5 demonstrate the statistical analysis for IHC staining and WB. *P < 0.05 and ^#^*P* < 0.05.

In the mouse H22 tumor model, the expression levels of CD4 and CD8 were higher in both groups B and C than in group A (*P* < 0.05), while in group D, the expression levels of CD4 and CD8 were significantly higher than in groups A and C (*P* < 0.05). (Fig. 4(1–4)). This indicated that the combined treatment had a synergistic effect and enhanced the activity of T cells. TGF-β showed similar changes (Fig. 4(5,6)). The expression levels of CD69, IL-6 and IFN-γ were higher in both groups B and C than in group A (*P* < 0.05), while in group D, the expression levels of CD69, IL-6 and IFN-γwere significantly higher than in groups A and C (*P* < 0.05). (Fig. 5(1–6)). The expression levels of PD-1 and PD-L1 were lower in group B and group C than in group A (*P* < 0.05); while in group D, the expression levels of PD-1 and PD-L1 were significantly lower than in groups A and C (*P* < 0.05) (Fig. 6(1–6)). This indicated that the combination therapy could improve the tumor microenvironment and promote immune activation.

### Safety and tolerability of the combination of CPT, apatinib, and PD-1 inhibitor

**Figure 7:**
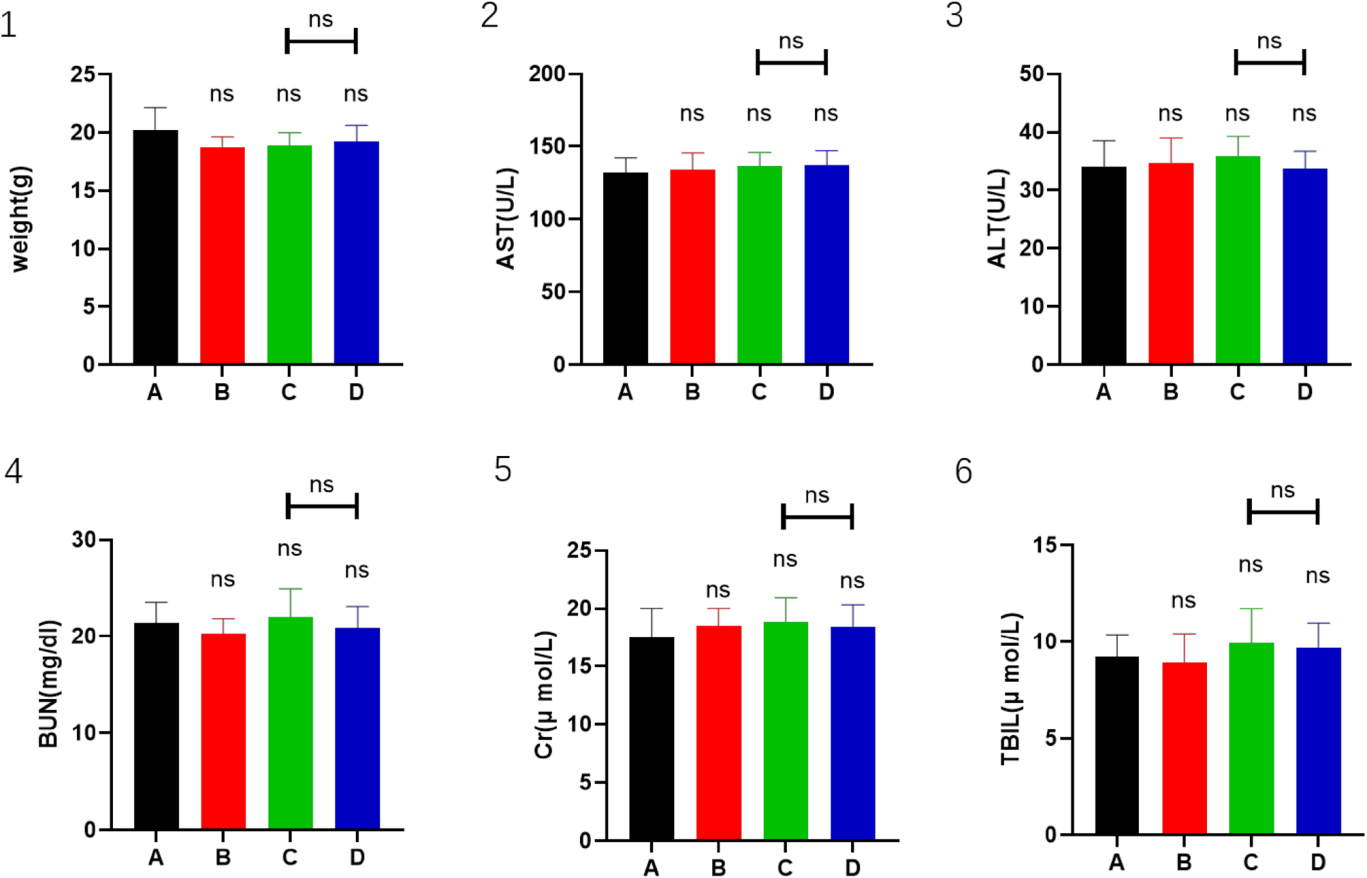
Panels A, B, C, D, E, and F show the statistical analysis of weight, AST, ALT, BUN, Cr, and TBIL.

There was no difference in body weight between mice in the four treatment groups after treatment and tumor resection. The body weights of the mice were 20.25 ± 1.79 g (A), 18.75 ± 0.83 g (B), 18.88 ± 1.05 g (C) and 19.25 ± 1.30 g (D) (*P* ≥ 0.05) (Fig. 7(1)). Serum analysis of mice showed that blood urea nitrogen, creatinine, total bilirubin, alanine transferase, and aspartate transferase were at normal levels without any treatment affecting liver and kidney functions (Fig. 7(2–6)).

## Discussion

PD-1 inhibitors in combination with anti-angiogenic drugs such as apatinib induce enhanced therapeutic effects and may become may be a promising strategy to extend the benefits of PD-1 inhibitor therapy to a wider group of hepatocellular carcinoma patients. However, there are many side effects associated with the combination of the two, and we expected to reduce the dosage of apatinib to make it more effective at a lower dose, so we applied CPT in combination to reduce the side effects and improve the anti-tumor effect. Our findings further suggest that CPT enhances the antitumor effects of low-dose apatinib combined with PD-1 inhibitors in HCC.

Tumor angiogenesis is essential for tumor growth and metastasis. VEGFA is the most important angiogenic factor in the vascular endothelial growth factor family, playing an important role in the occurrence and development of tumors. Studies have confirmed that high levels of VEGFA are related to poor prognosis in tumors such as in gastric cancer, ovarian cancer, HCC, non-small cell lung cancer, and endometrial cancer^39–42^. During tumor growth, high metabolism leads to a hypoxic microenvironment within the tumor, which activates growth factors and induces angiogenesis. Previous studies have shown that downregulation of Nrf2 reduces angiogenesis^43,44^, CPT also inhibited the vessel density^30^. Apatinib is a highly selective multiple TKI that very selectively blocks VEGFR2. It has been reported that apatinib effectively inhibits tumor proliferation and migration by blocking the VEGF axis^45^. Chen et al.^46^ reported that the inhibitory effect of apatinib on tumorigenesis may be bound up with the downregulation of VEGF and VEGFR2 expression in HCC. In this study, the expression levels of VEGFA and VEGFR2 in the CPT group were lower than those in the control group, confirming that CPT could inhibit the VEGFA signaling pathway.

Because of the pronounced pro-angiogenic effects of VEGFA, high VEGFA expression exacerbates tumor vascular abnormalities, poor perfusion, and inadequate oxygen supply. Hypoxic eventually regulates TME into an immunosuppressive environment^47,48^. Therefore, the hypoxia, angiogenesis, and immunosuppressive tumor microenvironment induced by VEGFA overexpression is definitely detrimental to PD-1 inhibitor^21^. A recent study demonstrated that low-dose apatinib combined with anti-pd-1 inhibitors optimizes the tumor microenvironment by alleviating hypoxia and promoting CD8 (+) T-cell infiltration^37^, which was reinforced in our study by the combined application of CPT. IL-6 modulates PD-L1 in tumor cells as well as modulating inflammatory and immune responses^49,50^. We noted that apatinib inhibited IL-6-mediated upregulation of PD-L1 in tumor cells. Apatinib at low doses significantly alleviated tumor hypoxia, increased CD4+ and CD8+ cell infiltration, and decreased TGF-β levels at certain time points, suggesting that angiogenesis inhibitors have a true immunomodulatory effect^37^. Studies by Schmittnaegel et al.^49^ and Elizabeth et al.^50^ provide evidence that antiangiogenic drugs specifically improve anti-PD-1/PD-L1 therapy when promoting an immunostimulatory tumor microenvironment and tumor vascular normalization in various tumor models. CD69 is the earliest cell surface marker for activated T cells, and we also found that apatinib promoted CD69 expression and IFN-γ secretion. Therefore, our results suggest that apatinib may partially restore the activation of T cells by targeting the VEGFR2/PD-L1 signaling pathway in tumor cells, thus exerting its tumor suppressive effect, which was likewise enhanced by the combined application of CPT. In this study, CPT was confirmed to enhance the antitumor effect of low-dose apatinib combined with PD-1 inhibitor in HCC.

CPT was proved as a potent Nrf2 inhibitor among multitudinous agents^34^. CPT has a proven safety profile and is used clinically for chemotherapy drug^51,52^. In our previous studies, CPT was shown to be effective in inhibiting ROS levels and Nrf2 expression^30,34^. Increasing evidence suggests that Nrf2 plays a important role in autophagy regulation by forming positive feedback with p62. It has been proved that insufficient autophagy leads to p62 accumulation, which further segregates Keap1, a negative regulator of Nrf2, leading to Nrf2 stabilization^53^. Apatinib induces cellular autophagy and apoptosis by promoting ROS generation and inhibiting Nrf2 and p62 expression^5^. This study confirms that the combination treatment can downregulate Nrf2 by inhibiting the Nrf2 axis, which is beneficial for inhibiting the growth of HCC as well as inducing autophagy and apoptosis in tumor cells. Notably, our work is limited to some extent by the ability of animal models to mimic human TME and the hypothesis-generating nature of the study, which suggests the need for further evaluation in more mouse experimental systems.

In conclusion, this study confirms that CPT enhances the antitumor effect of low-dose apatinib combined with PD-1 inhibitors in HCC.

### Supplementary Information

Supplementary Information.

## Data Availability

Data is provided within the manuscript or supplementary information files.
