# Author Correction: Camptothecin enhances the anti-tumor effect of low-dose apatinib combined with PD-1 inhibitor on hepatocellular carcinoma

**DOI:** 10.1038/s41598-024-59647-7

**Published:** 2024-04-16

**Authors:** Hankang Wang, Congcong Gao, Xiaodong Li, Feng Chen, Guijie Li

**Affiliations:** 1 Department of Radiology, The First Affiliated Hospital of Shandong First Medical University, Jinan, Shandong 250000 People’s Republic of China; 2 Jinan Center for Disease Control and Prevention, Jinan, Shandong 250000 People’s Republic of China; 3 Department of Radiology, The First Affiliated Hospital of Shandong First Medical University, Jinan, Shandong 250000 People’s Republic of China; 4 Department of Radiology, The First Affiliated Hospital of Shandong First Medical University and Shandong Provincial Qianfoshan Hospital, 16766 Jingshi Road, Lixia, Jinan, Shandong 250014 People’s Republic of China

Correction to: *Scientific Reports* 10.1038/s41598-024-57874-6, published online 26 March 2024

In the original version of this Article Guijie Li was incorrectly affiliated with ‘Jinan Center for Disease Control and Prevention, Jinan, Shandong 250000, People’s Republic of China’. The correct affiliation for Guijie Li is listed below.

Department of Radiology, The First Affiliated Hospital of Shandong First Medical University and Shandong Provincial Qianfoshan Hospital, 16766 Jingshi Road, Lixia, Jinan, Shandong 250014, People’s Republic of China.

The original Article has been corrected.

