# Supplementary figures and images for: Camptothecin enhances the anti-tumor effect of low-dose apatinib combined with PD-1 inhibitor on hepatocellular carcinoma
Source: Sci Rep. 2024 Mar 26;14:7140. doi: 10.1038/s41598-024-57874-6 (PMC10966085; doi:10.1038/s41598-024-57874-6)

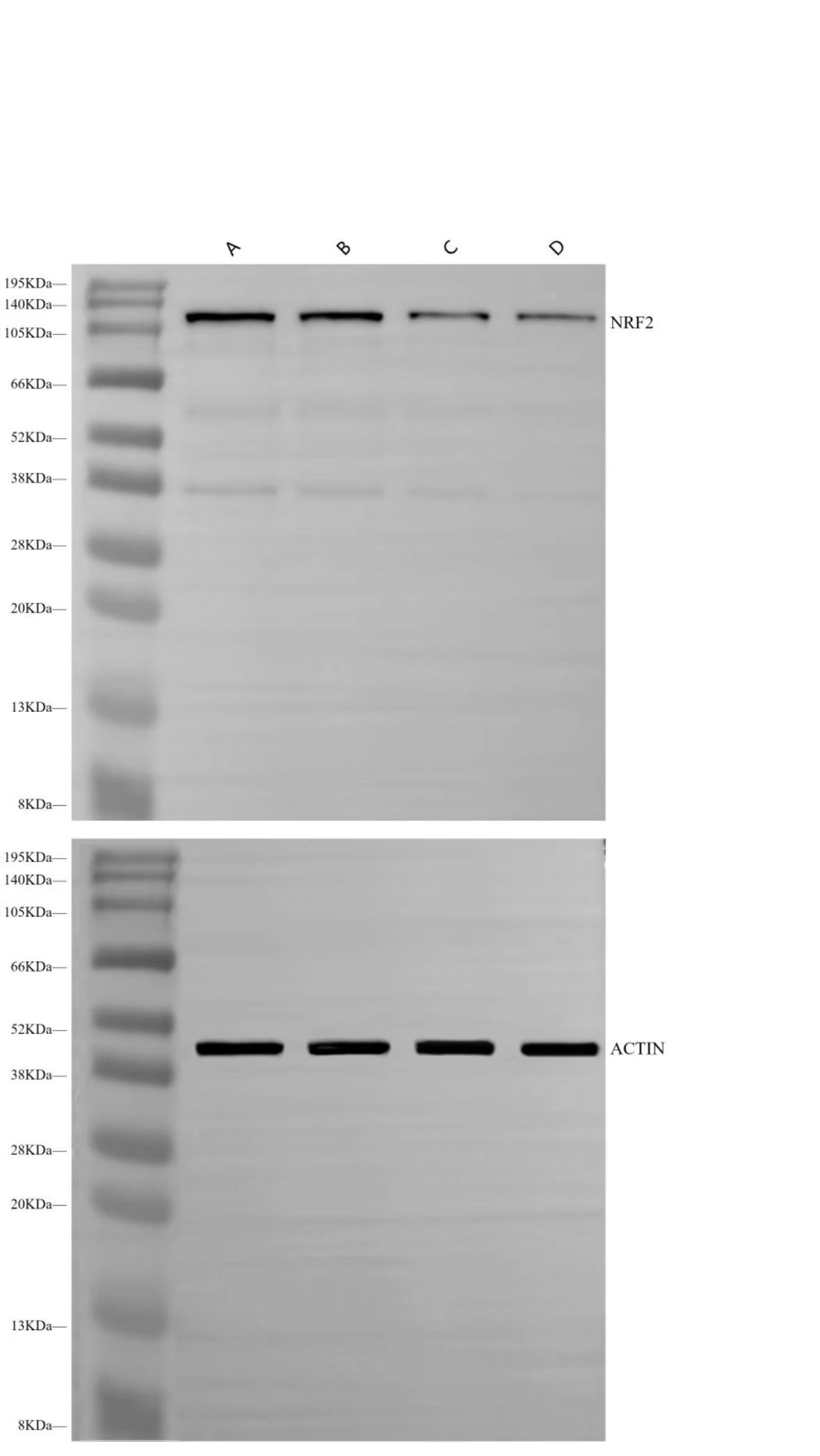

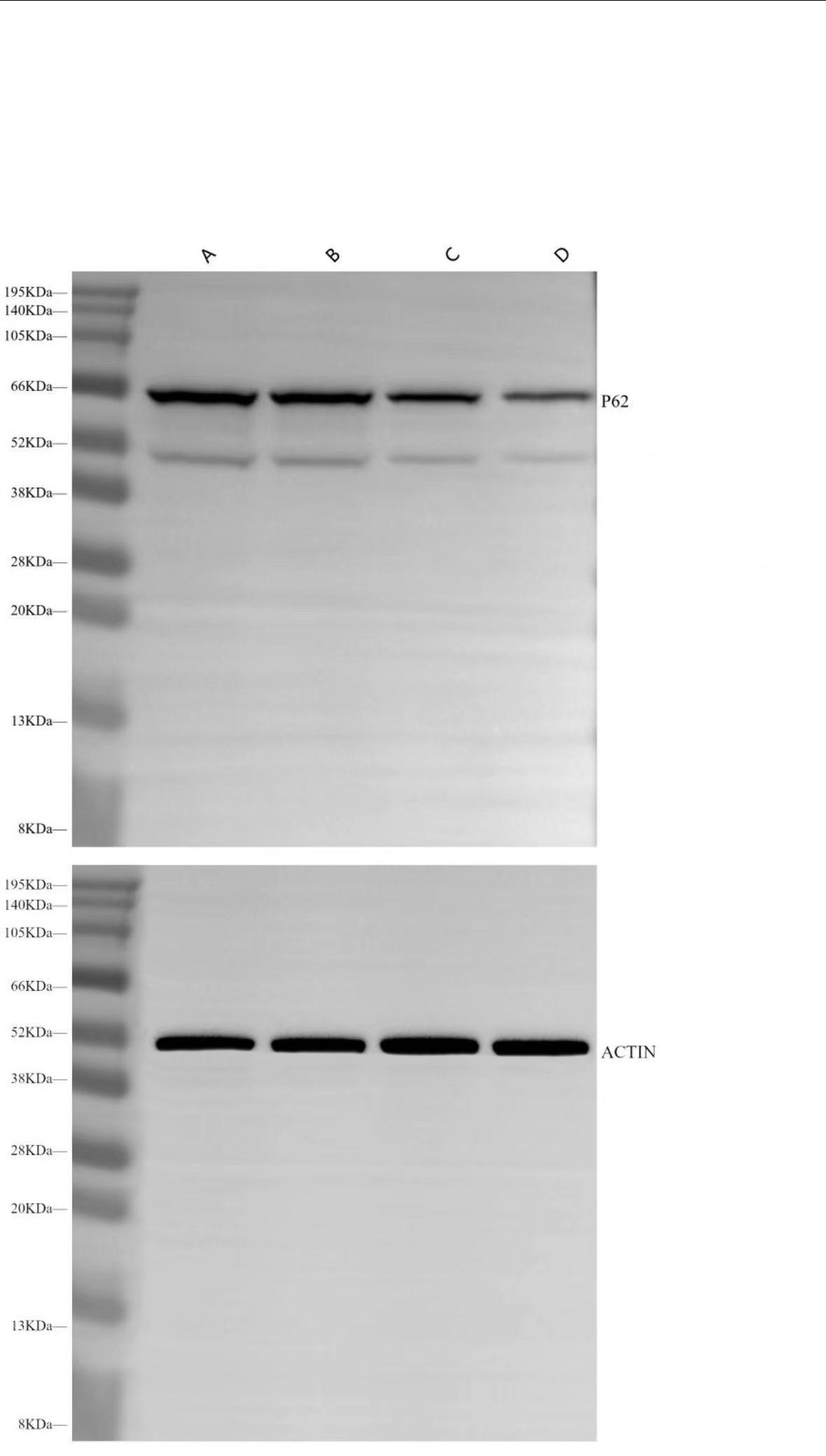

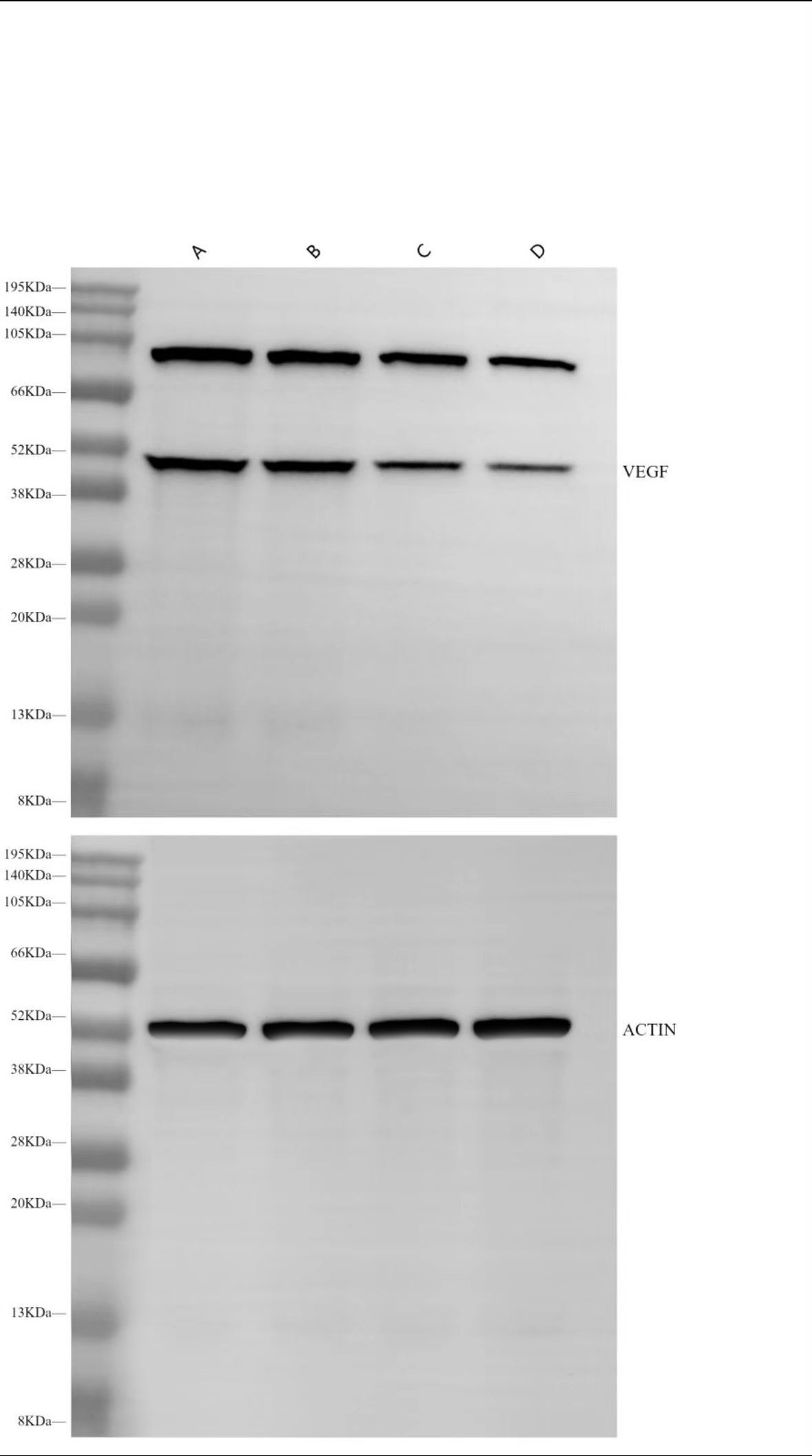

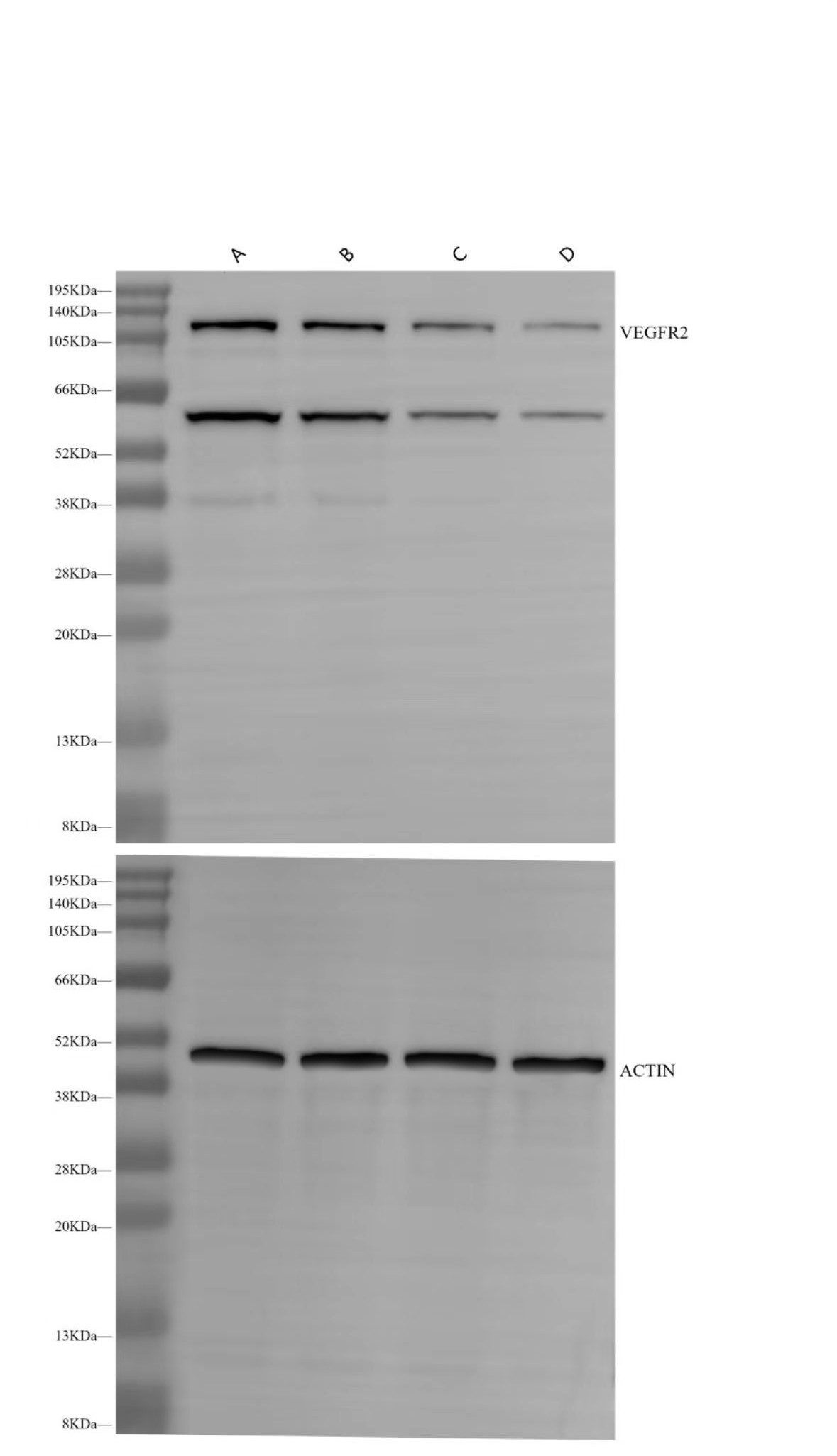

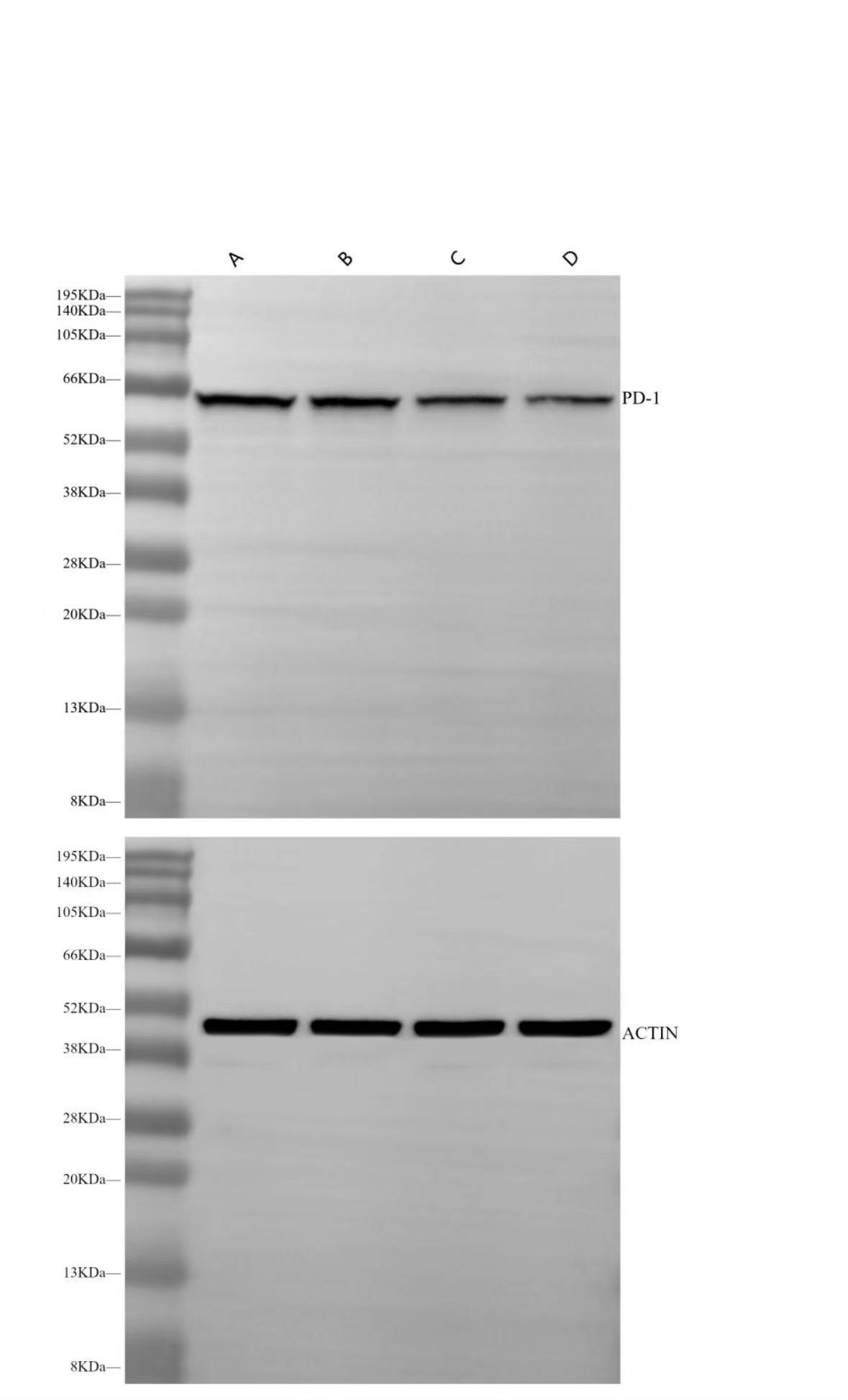

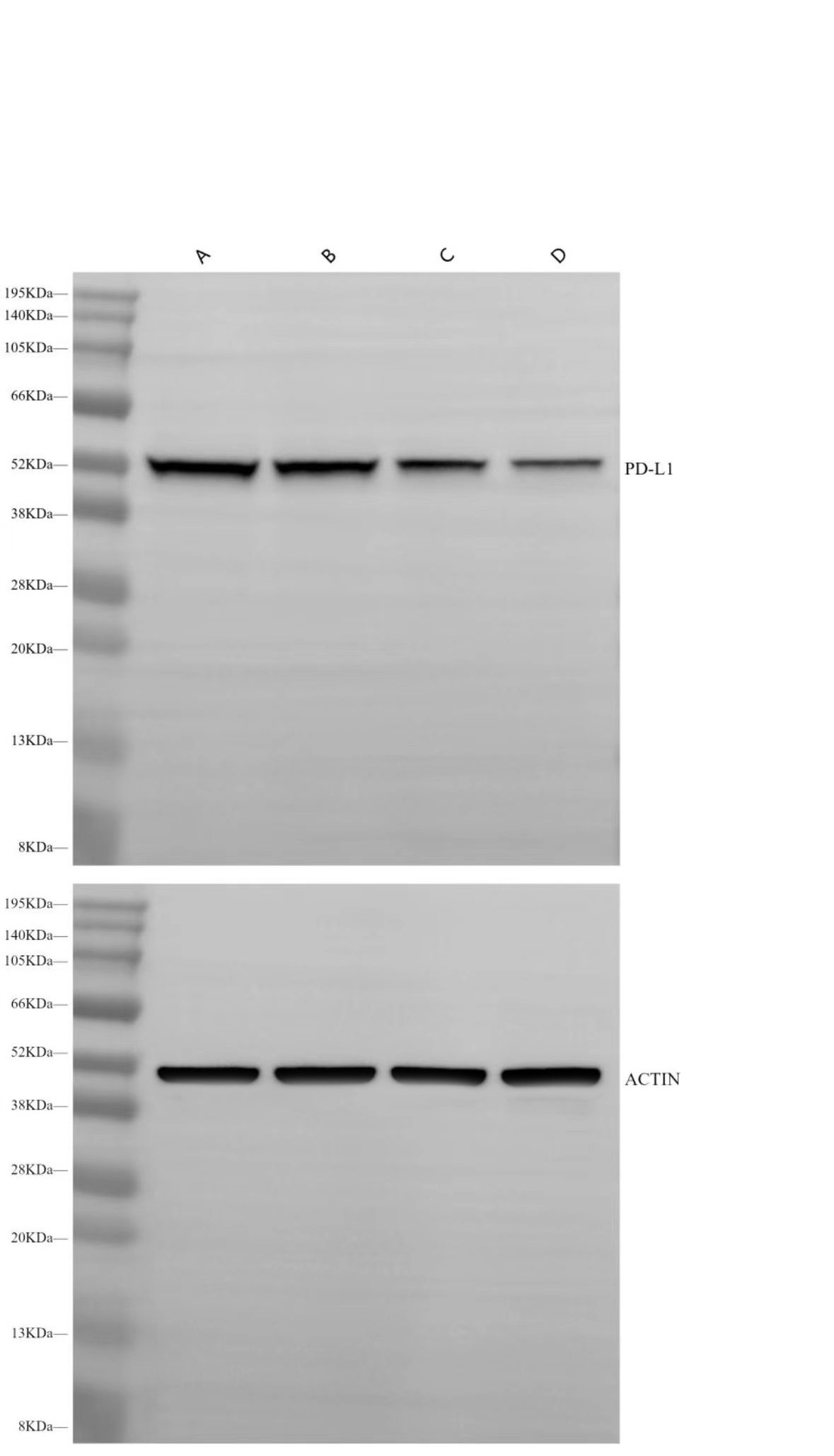


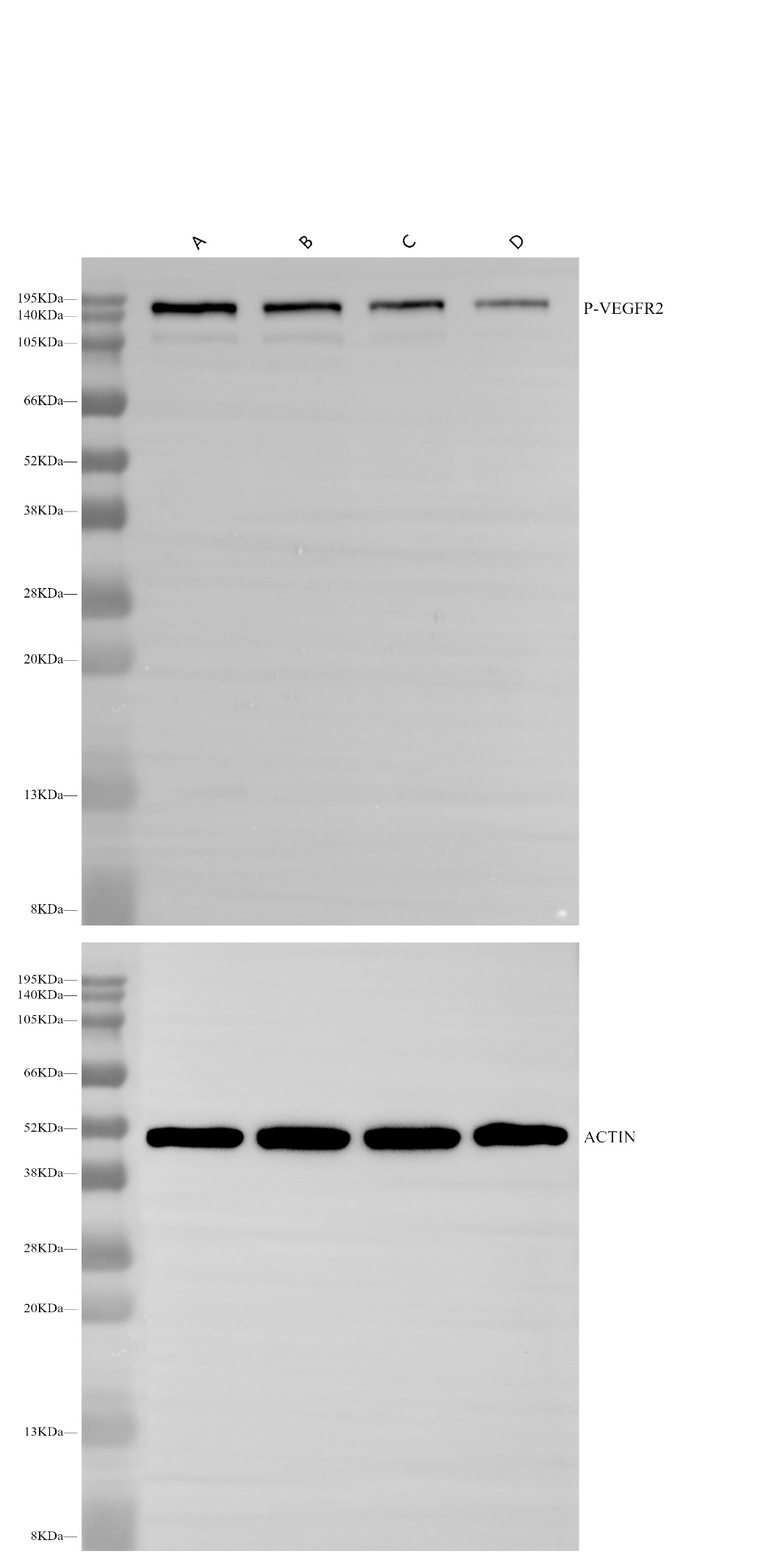

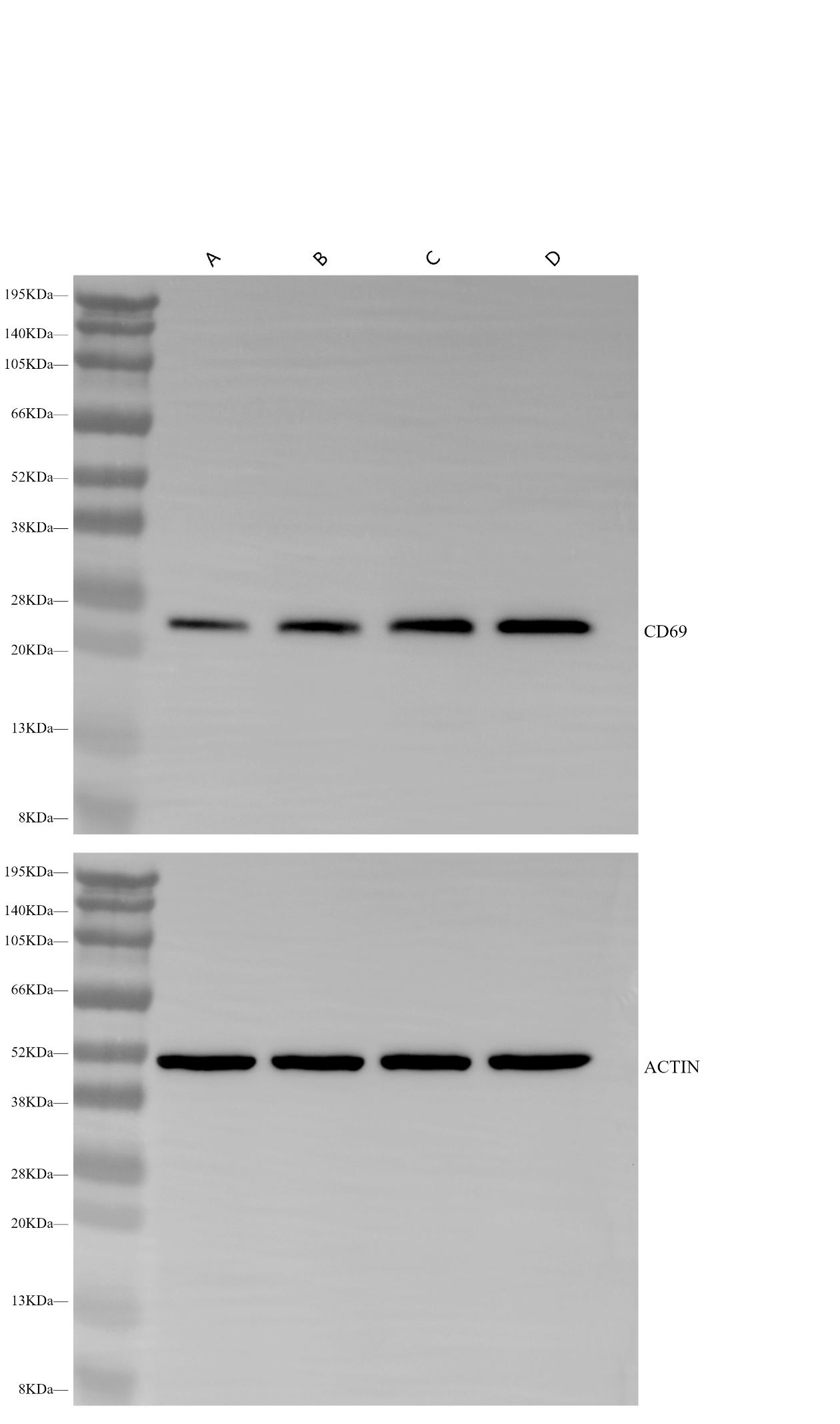

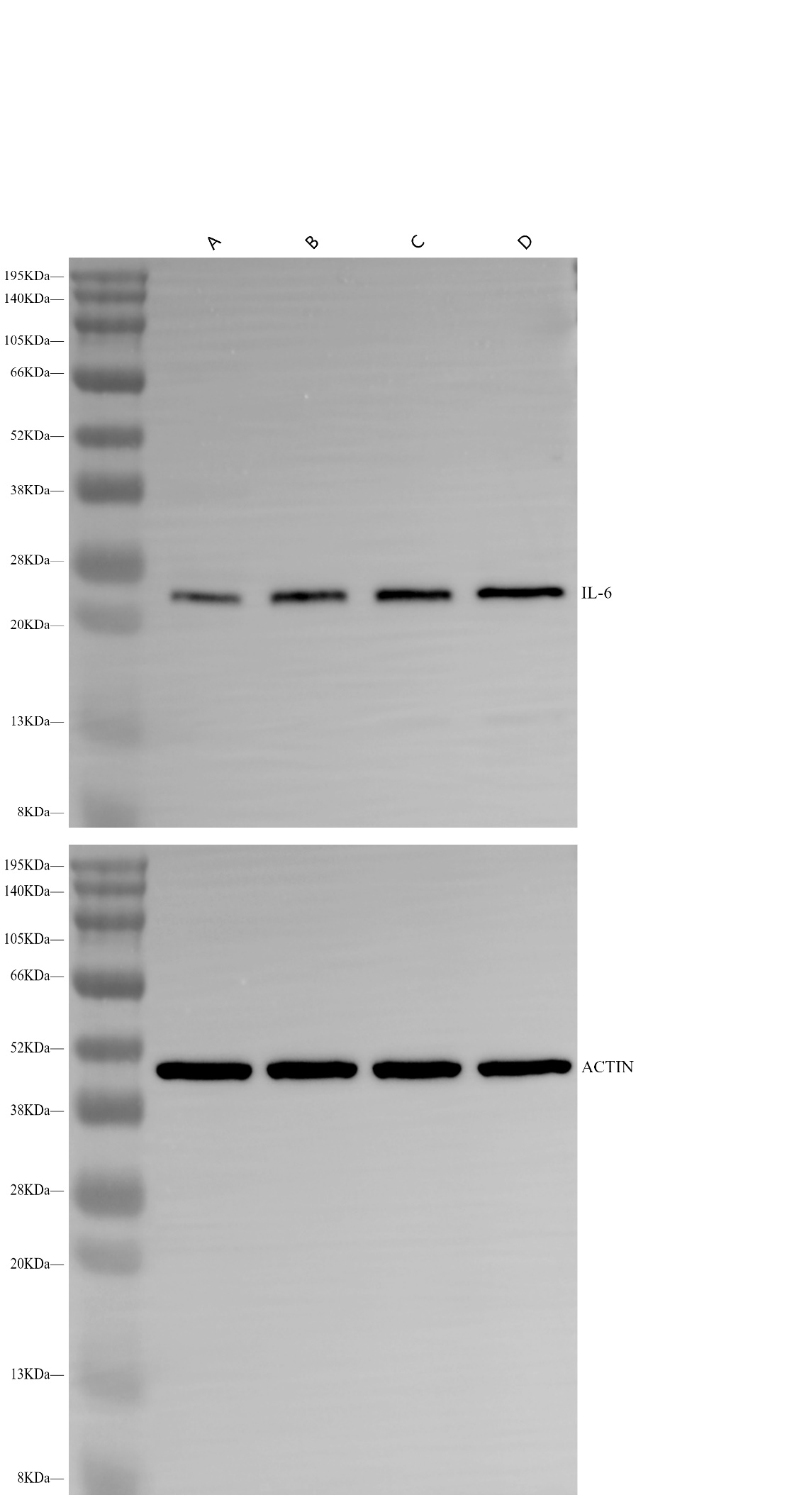

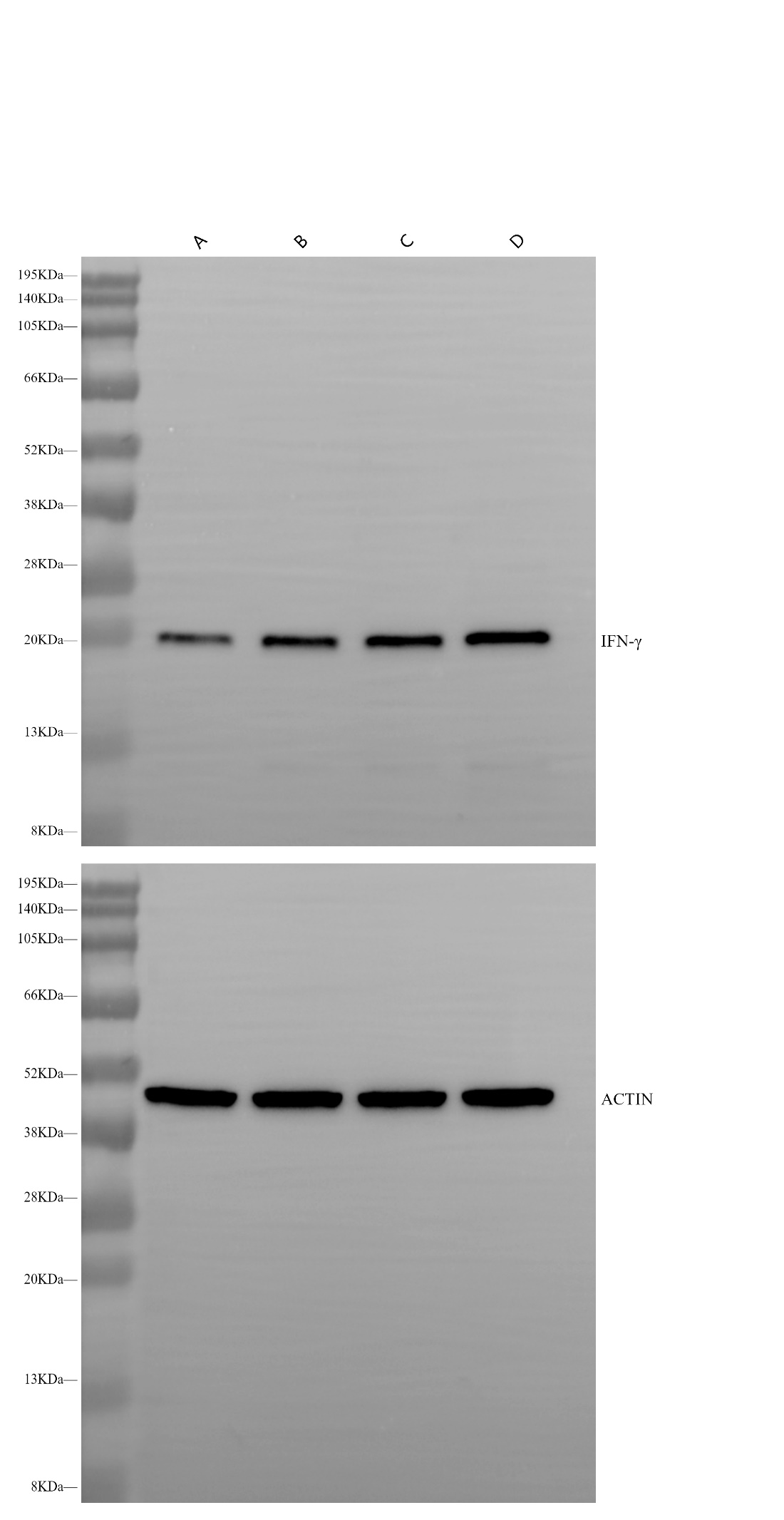

Supplement: Supplementary file 1 — Supplementary Information. [file 41598_2024_57874_MOESM1_ESM.docx]
